# Early alveolar molecular signatures after cardiopulmonary resuscitation: a bronchoalveolar lavage (BALF) proteomic study in swine

**DOI:** 10.1016/j.resplu.2026.101405

**Published:** 2026-07-06

**Authors:** Joaquin Araos, Felipe Teran, Clark Owyang, Derek Lao, Congli Zeng, Marcos Vidal Melo, Qin Fu, Rory C. Chien, Michael Garenani, Manuel Martin-Flores, Marta Cercone

**Affiliations:** a Department of Clinical Sciences, College of Veterinary Medicine, Cornell University, Ithaca, NY, United States; b Department of Emergency Medicine, New York-Presbyterian Hospital/Weill Cornell Medical College, New York, NY, United States; c Division of Pulmonary and Critical Care Medicine, Department of Medicine, New York-Presbyterian Hospital/Weill Cornell Medical College, New York, NY, United States; d Department of Anesthesiology, University of Texas Medical Branch, Galveston, TX, United States; e Proteomics & Metabolomics Facility, Cornell Institute of Biotechnology, Cornell University, Ithaca, NY, United States; f Department of Population Medicine and Diagnostic Sciences, College of Veterinary Medicine, Cornell University, Ithaca, NY, United States

**Keywords:** Cardiopulmonary resuscitation, Acute respiratory distress syndrome, Bronchoalveolar lavage fluid, Proteomics, Lung injury

## Abstract

**Background:**

Mechanical chest compressions during cardiopulmonary resuscitation (CPR) can produce pulmonary edema, described as cardiopulmonary resuscitation–associated lung edema (CRALE), which has been largely interpreted as a hydrostatic phenomenon. However, whether early molecular changes associated with epithelial injury, coagulation, and inflammation are also present during resuscitation remains uncertain. We hypothesized that bronchoalveolar lavage fluid proteomics would identify early protein signatures of lung injury after CPR.

**Methods:**

Bronchoalveolar lavage fluid (BALF) was collected separately from the right and left caudal lung lobes of nine pigs before cardiac arrest and again after ventricular fibrillation, 8 min of untreated arrest, and up to 45 min of CPR. In animals achieving return of spontaneous circulation, post-CPR BALF was obtained immediately after resuscitation; in the remaining animals, sampling was performed at the end of CPR. Proteins were digested, tandem mass tag 18-plex labeled, and analyzed by high-resolution liquid chromatography–mass spectrometry. Differentially abundant proteins were defined by fold change >1.5 or <0.67 with *P* < 0.05. Functional enrichment, histologic assessment, and western blot validation were performed.

**Results:**

A total of 1088 proteins were identified in left lung BALF and 1200 in right lung BALF. Comparison of pre- and post-CPR samples identified 74 and 78 differentially abundant proteins in the left and right lungs, respectively, with 28 shared proteins across both lungs. Among the most increased proteins were plasminogen activator inhibitor-1 (SERPINE1/PAI-1), apolipoprotein A1, inter-alpha inhibitor family proteins, vitronectin, histones, and advanced glycation end-product receptor (AGER). Functional enrichment showed over-representation of proteins related to complement and coagulation, platelet degranulation, lipid metabolism, and extracellular matrix signaling. Histology showed mild septal thickening, vascular neutrophilic infiltration, interstitial edema, and patchy fibrin deposition, consistent with early pneumocyte injury and mild inflammation. Western blot confirmed increased post-CPR abundance of PAI-1 and apolipoprotein A1.

**Conclusions:**

Early after resuscitation, BALF proteomics identified molecular changes characterized by increased antifibrinolytic and complement-related proteins, together with proteins associated with epithelial and endothelial stress. These findings suggest that pulmonary abnormalities after CPR may involve not only hydrostatic edema but also an early biologic response consistent with lung injury, which may contribute to post-cardiac arrest respiratory dysfunction and progression to acute respiratory distress syndrome.

## Introduction

Cardiac arrest is frequently followed by post–cardiac arrest syndrome (PCAS), a systemic ischemia–reperfusion injury associated with inflammation, oxidative stress, and multiorgan dysfunction.^1^ Pulmonary complications are common after successful resuscitation, and respiratory failure contributes substantially to morbidity and mortality in cardiac arrest survivors. Imaging studies report lung abnormalities in up to 100% of patients after cardiopulmonary resuscitation (CPR),^2^, ^3^ and acute respiratory distress syndrome (ARDS) is a frequent and clinically important complication in this population.^4^, ^5^

The mechanisms responsible for post–cardiac arrest lung injury remain uncertain. Experimental work by Magliocca and colleagues introduced the concept of cardiopulmonary resuscitation–associated lung edema (CRALE), suggesting that pulmonary injury after CPR may be predominantly hydrostatic and related to the mechanical and hemodynamic effects of chest compressions, with limited evidence of early inflammatory or permeability-mediated damage.^6^ This model has supported the view that lung injury after resuscitation is primarily mechanical in origin and develops independently of the biological pathways typically associated with ARDS.

However, the high incidence of ARDS after cardiac arrest raises the possibility that permeability-driven injury begins earlier than currently recognized.^7^ Because ARDS is defined by disruption of the alveolar–capillary barrier, activation of coagulation and innate immune pathways, and epithelial stress, an important unanswered question is whether these molecular processes are already initiated during resuscitation itself.

We therefore performed unbiased proteomic analysis of bronchoalveolar lavage fluid (BALF) collected before and immediately after CPR in a clinically relevant porcine cardiac arrest model. We hypothesized that resuscitation would be associated with rapid activation of molecular pathways linked to barrier dysfunction, coagulation, and innate immune signaling, consistent with early ARDS-like biology occurring at the time of resuscitation rather than developing later during intensive care.

## Materials and methods

### Experimental protocol

Detailed animal and experimental procedures are described in supplementary file 1. Briefly, all procedures were conducted in accordance with the ARRIVE guidelines^8^ and were approved by the Cornell University’s Institutional Animal Care and Use Committee (protocol 2022-0135). Nine juvenile pigs (4 male, 5 female; body weight 50 ± 6 kg) were anesthetized and mechanically ventilated in volume-controlled mode with a tidal volume of 8 mL/kg, respiratory rate 12–14 breaths/min, positive end-expiratory pressure of 5 cmH_2_O, and inspired oxygen fraction of 0.6.

After vascular access was established, a pulmonary artery catheter and pacing wire were placed to allow hemodynamic monitoring and induction of ventricular fibrillation (VF). VF was induced electrically as previously described.^9^ Following VF induction, animals underwent 8 min of untreated cardiac arrest, after which mechanical cardiopulmonary resuscitation (CPR) and positive-pressure ventilation were initiated. No drugs or defibrillation were administered during the first 5 min of CPR. Advanced cardiac life support was then initiated with epinephrine every 3 min and defibrillation after 10 min of CPR. Resuscitation efforts continued until return of spontaneous circulation (ROSC) or for a maximum of 45 min (see Supplementary Fig. 1 for representative recorded traces of the experimental events).

### Bronchoalveolar lavage

BALF was collected bronchoscopically in separate samples from the left and right caudal lung lobes before cardiac arrest and again within 45 min after cardiac arrest and CPR, or immediately after return of spontaneous circulation when achieved. Samples were immediately centrifuged, supplemented with protease inhibitors, and stored at −80°C until analysis. A detailed description of BALF collection and processing is provided in Supplementary File 1.

### Proteomic analysis

Detailed descriptions of protein extraction, digestion, TMT18-plex labeling, liquid chromatography-mass spectrometry, database searching, and quantitative processing are provided in Supplementary File 1. Briefly, BALF samples from the left and right lungs were analyzed separately using TMT18-plex quantitative proteomics and nanoLC-MS/MS. Differential abundance analysis comparing post-CPR versus pre-CPR samples was performed independently for each lung. Principal component analysis and volcano plots were generated separately for the left and right lungs, after which overlapping differentially abundant proteins common to both lungs were retained for downstream analyses, including heatmaps, protein–protein interaction networks, gene ontology enrichment, and pathway analysis.

### Western blot validation

Western blot analysis was performed to confirm selected proteomic findings for APOA1 and PAI-1, which were among the most increased proteins identified after CPR. BALF samples were pooled by time point and lung, and equal amounts of protein were analyzed by SDS-PAGE and immunoblotting. Band intensity was quantified by densitometry, normalized to total protein staining, and expressed as post-CPR to pre-CPR ratios for each lung. A detailed description of western blot procedures is provided in Supplementary File 1.

### Histologic analysis

Lung tissue from three animals was collected at the end of the experiment from caudal lung regions corresponding to the BALF sampling sites for histologic evaluation. Samples were formalin-fixed, paraffin-embedded, sectioned, and stained with hematoxylin-eosin and phosphotungstic acid hematoxylin to assess structural changes and fibrin deposition. Histologic evaluation was performed by a blinded board-certified veterinary pathologist. A detailed description of tissue processing and histologic analysis is provided in Supplementary File 1.

## Results

BAL procedure was conducted without complications on all nine pigs before inducing cardiac arrest and after CPR. Five of nine pigs did not achieve ROSC, and post-CPR BALF was therefore collected 45 min after the start of CPR. In the remaining four pigs, ROSC was achieved after 25, 28, 36, and 36 min of resuscitation (average 31 min), and post-CPR BALF was collected immediately thereafter. No-flow duration was fixed at 8 min in all animals, and epinephrine timing, defibrillation timing, and ventilation followed a standardized protocol. End-tidal CO_2_ and systolic arterial pressure during the 5-minute window preceding ROSC, or preceding the end of CPR in non-resuscitated animals, were comparable between groups (end-tidal CO_2_ 24 ± 9 versus 22 ± 7 mmHg; systolic arterial pressure 56 ± 18 versus 56 ± 23 mmHg).

### Proteomic analysis

#### Proteomics alterations after CPR

Warm saline (2 mL/kg per lobe) was instilled and retrieved, with a mean recovery of approximately 75% that was consistent across animals and time points. Total BALF protein concentration increased after CPR in both lungs (left, 2.3 ± 1.0–3.4 ± 1.9 mg/mL; right, 2.2 ± 1.1–3.5 ± 1.6 mg/mL; combined paired *P* = 0.015). Because lavage recovery was unchanged, this increase reflects greater alveolar protein content after resuscitation rather than differences in recovery or dilution.

A total of 1088 proteins from the left lung BALF and 1200 from the right lung BALF were identified by quantitative proteomics analysis. Based on statistical analysis, 74 differentially abundant proteins (DAPs) with fold change (FC) >1.5 or <0.67 and *P* < 0.05 were identified in the left lung BALF between the pre- and post-CPR samples, including 45 with increased abundance and 29 with decreased abundance. In the right lung BALF, 78 DAPs met these criteria, including 49 with increased abundance and 29 with decreased abundance.

**Fig. 1:**
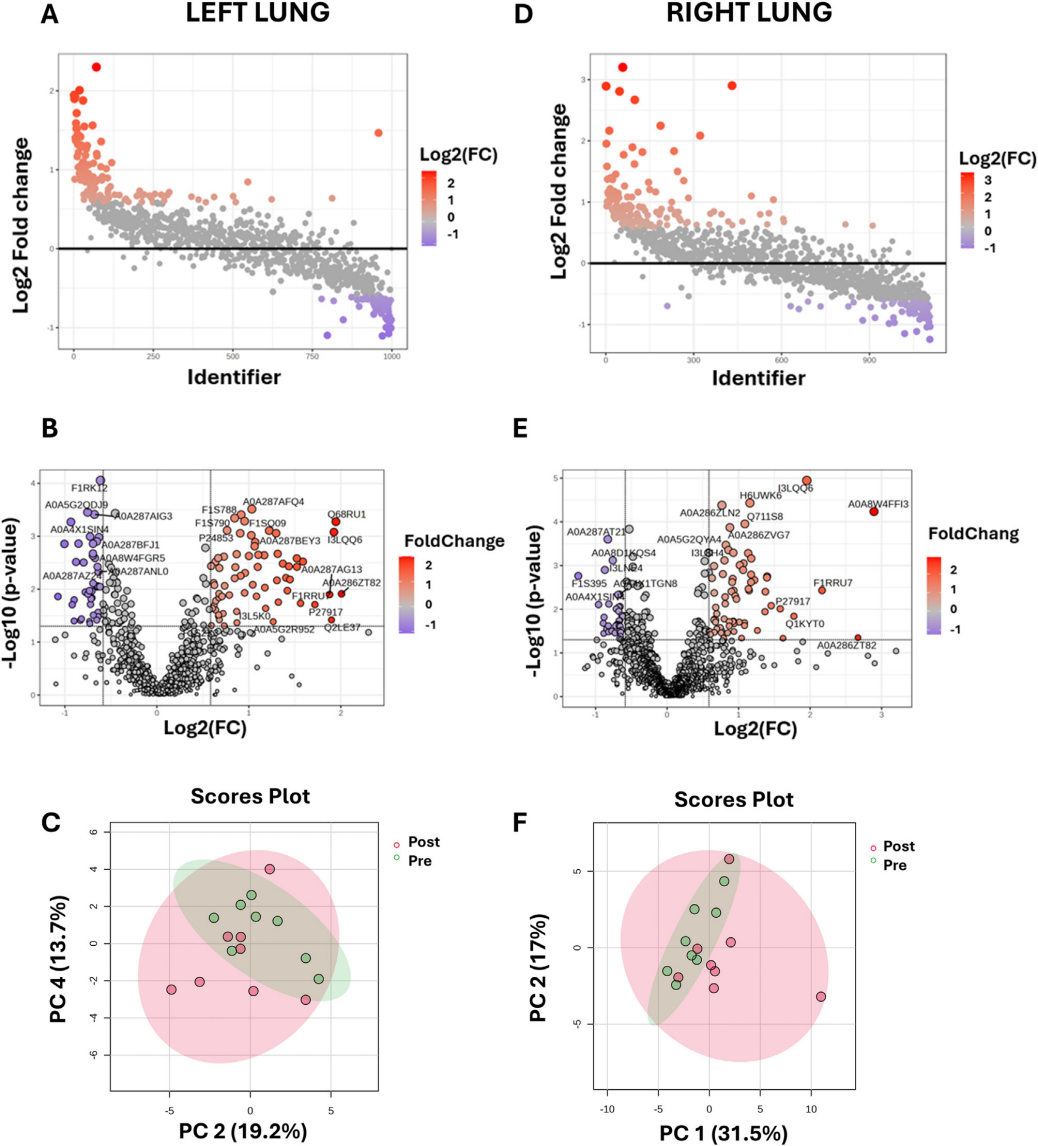
**Proteomic profiling of bronchoalveolar lavage fluid (BALF) before (Pre) and after cardiopulmonary resuscitation (Post) in the left and right lungs**. Panels A and D show ranked fold-change plots for all identified proteins in the left and right lungs, respectively, with proteins colored according to log2 fold change (Log2FC), highlighting proteins increased (red) or decreased (purple) after CPR relative to baseline. Panels B and E show volcano plots of differential protein abundance for the left and right lungs, respectively, with significantly increased and decreased proteins identified using fold-change and statistical significance thresholds and selected proteins labeled. In the left lung, 74 differentially abundant proteins were identified, while the right lung showed a similar number of significant changes. Panels C and F show PLS-DA score plots demonstrating partial separation between Pre and Post samples in the left and right lungs, respectively, indicating a CPR-associated shift in the BALF proteomic profile. (For interpretation of the references to color in this figure legend, the reader is referred to the web version of this article.)

Fold changes, volcano plots and principal component analysis for each lung are shown in Fig. 1. Complete lists of identified proteins, quantified proteins, differentially abundant proteins, overlapping DAPs between lungs, and gene ontology annotations for the left and right lungs are provided in Supplementary File 2.

#### Shared injury and inflammatory signatures in both lungs following CPR

**Fig. 2:**
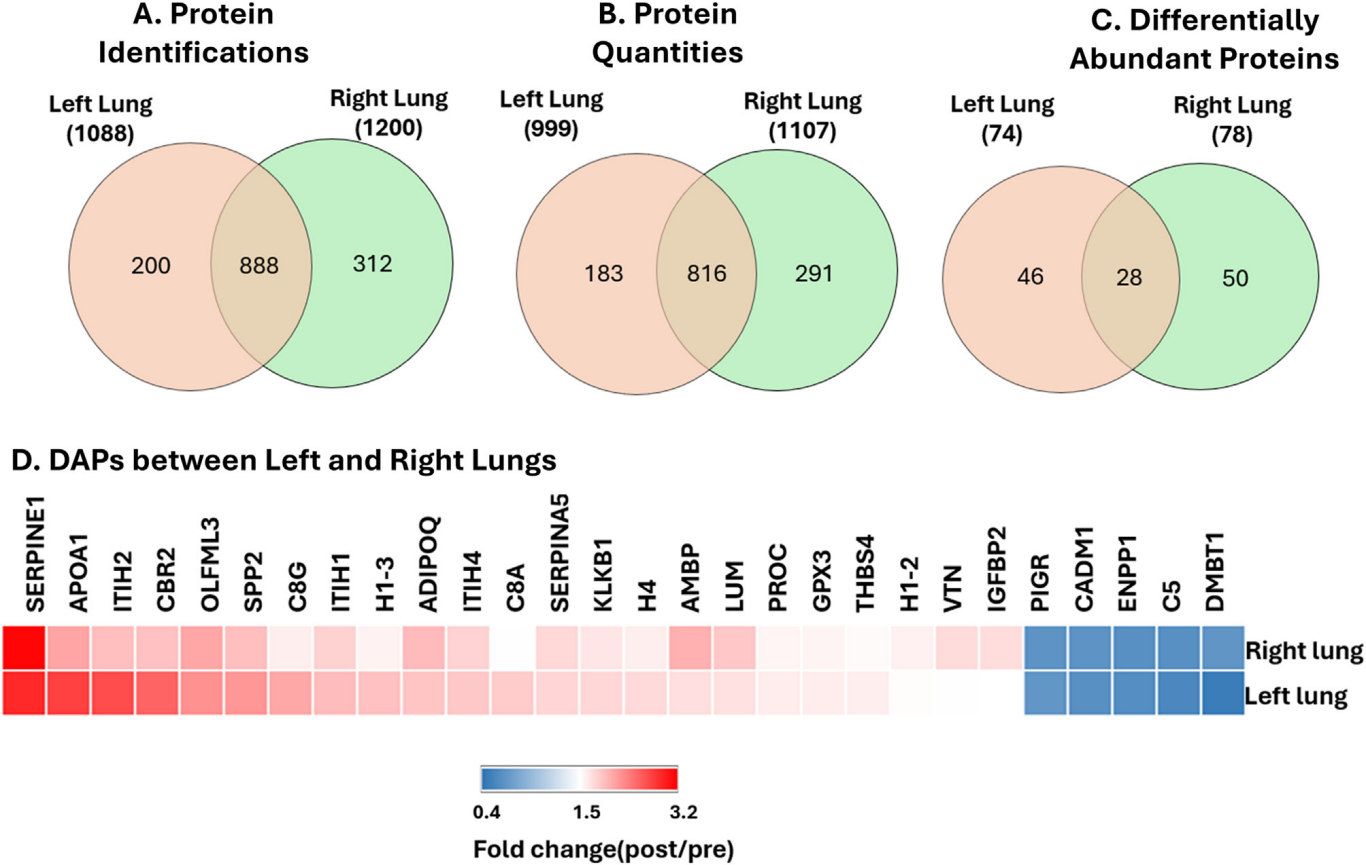
**Overlap of protein identifications and differentially abundant proteins (DAPs) between the left and right lungs following cardiopulmonary resuscitation**. Panel A shows the total number of proteins identified in bronchoalveolar lavage fluid (BALF), with 1088 proteins identified in the left lung and 1200 in the right lung, including 888 shared proteins. Panel B shows quantified proteins used for comparative analysis, with 816 proteins shared between lungs. Panel C shows overlap of DAPs identified after CPR, with 74 DAPs in the left lung and 78 in the right lung, including 28 shared proteins. Panel D shows the fold-change heatmap of the 28 shared DAPs between lungs, with proteins increased after CPR shown in red and decreased proteins shown in blue. Serpin family E member 1 (SERPINE1, plasminogen activator inhibitor-1 [PAI-1]) showed the greatest increase in both lungs, along with increases in apolipoprotein A1 (APOA1), inter-alpha-trypsin inhibitor heavy chain (ITIH) family proteins, histones, vitronectin (VTN), and advanced glycosylation end-product specific receptor (AGER). Decreased proteins common to both lungs included polymeric immunoglobulin receptor (PIGR), cell adhesion molecule 1 (CADM1), ectonucleotide pyrophosphatase/phosphodiesterase 1 (ENPP1), complement C5 (C5), and deleted in malignant brain tumors 1 (DMBT1). (For interpretation of the references to color in this figure legend, the reader is referred to the web version of this article.)

To explore the commonality of lung responses to CPR, we analyzed overlapping DAPs identified in BALF collected from the left and right lungs. We identified 28 shared DAPs, including 23 upregulated and 5 downregulated proteins in both lungs (Fig. 2). In an exploratory analysis stratified by ROSC status, all 28 shared proteins changed in the same direction in animals that achieved ROSC and those that did not, in both lungs, and principal component analysis colored by ROSC status showed no separation between subgroups (Supplementary Fig. 2), indicating that the shared signature was not driven by ROSC status. Among these, SERPINE1 (PAI-1), a glycoprotein involved in fibrinolysis, cell migration, revascularization, inflammatory reactions, and tissue repair, exhibited one of the largest fold changes after CPR.^10^ Several histones, including histone H4 and H1 variants (H1-2, H1-3), known mediators of tissue injury and organ dysfunction,^11^, ^12^ were also significantly increased in BALF from both lungs. Thrombospondin-4 (THBS4), an extracellular matrix protein associated with vascular inflammation and angiogenesis^13^, ^14^ was similarly elevated in both lungs after CPR.

Additional proteins associated with epithelial injury, including AGER (sRAGE), a marker of type I alveolar epithelial cell injury,^15^ were increased in individual lung analyses but were not part of the overlapping DAP set used for downstream heatmap and pathway analyses (Supplementary File 2). Other shared DAPs included lumican (LUM), a proteoglycan involved in extracellular matrix organization and collagen assembly, and glutathione peroxidase 3 (GPX3), an extracellular antioxidant enzyme. Both proteins showed mild increases in abundance after CPR in the left and right lungs. Among the shared downregulated proteins were PIGR, CADM1, ENPP1, C5, and DMBT1, which are involved in mucosal immunity, epithelial cell adhesion, innate host defense, and epithelial barrier formation.

#### Potential endogenous protective mechanisms in both lungs following CPR

In addition to the injury and inflammatory signatures identified among the shared DAPs, several proteins suggested activation of endogenous protective responses after CPR. Vitronectin (VTN), an extracellular matrix protein that interacts with PAI-1 and regulates cell adhesion, migration, proliferation, and survival was increased in both lungs.^16^ Members of the inter-alpha inhibitor family, including ITIH1, ITIH2, ITIH4, and AMBP, which can neutralize histone-associated cytotoxicity,^17^ were also significantly increased. In addition, APOA1 (apolipoprotein A-I), the major protein component of high-density lipoprotein with known anti-inflammatory and antioxidant properties, was among the most upregulated shared proteins after CPR.^18^

#### Bioinformatics functional characterization of DAPs

**Fig. 3:**
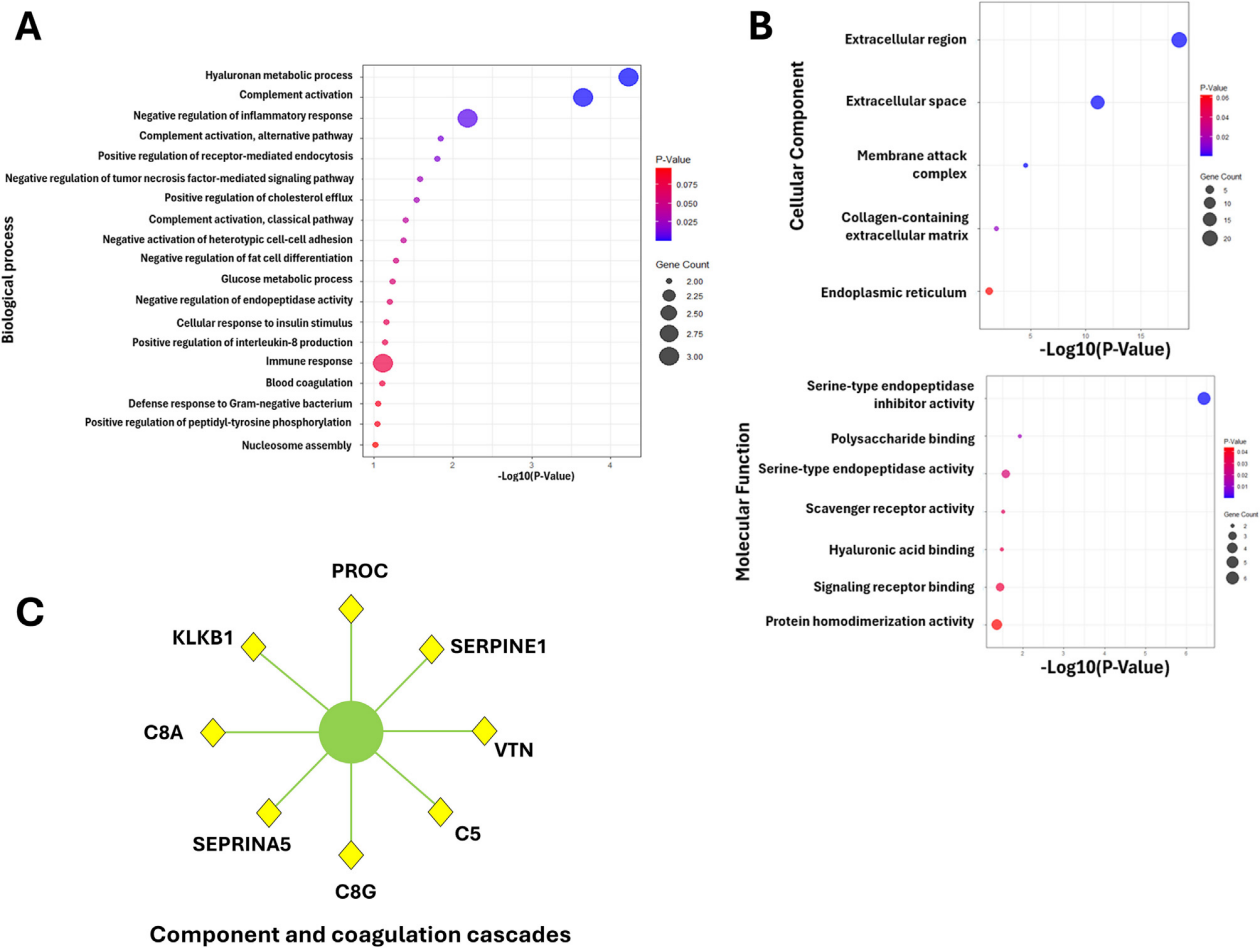
**Functional enrichment analysis of shared differentially abundant proteins identified in bronchoalveolar lavage fluid after cardiopulmonary resuscitation**. Panel A shows Gene Ontology biological process enrichment, with the most represented pathways including hyaluronan metabolic process, complement activation, negative regulation of inflammatory response, immune response, blood coagulation, and defense response to Gram-negative bacteria. Panel B shows Gene Ontology cellular component and molecular function enrichment, highlighting extracellular region, extracellular space, membrane attack complex, collagen-containing extracellular matrix, and endoplasmic reticulum, as well as serine-type endopeptidase inhibitor activity, serine-type endopeptidase activity, scavenger receptor activity, and hyaluronic acid binding. Panel C shows the protein interaction network of the complement and coagulation cascade pathway, centered around proc protein C (PROC), with interactions involving serpin family E member 1 (SERPINE1, plasminogen activator inhibitor-1 [PAI-1]), vitronectin (VTN), complement components C5, C8A, and C8G, kallikrein B1 (KLKB1), and serpin family A member 5 (SERPINA5).

To functionally characterize the shared DAPs identified in both lungs, gene ontology analysis was performed across three functional categories including biological processes, cellular components, and molecular functions (Fig. 3A). Enriched biological processes included complement activation, innate immune response, blood coagulation, extracellular matrix organization, and acute phase response. Cellular component analysis showed predominant enrichment in extracellular region and plasma proteins. Molecular function analysis demonstrated enrichment of serine-type endopeptidase inhibitor activity, protein binding, and calcium ion binding.

Protein–protein interaction analysis of the 28 shared DAPs revealed a central interaction network including C5, C8A, C8G, VTN, ITIH1, ITIH2, ITIH4, SPP2, APOA1, and AMBP (Fig. 3B). Pathway enrichment analysis further demonstrated significant overrepresentation of complement and coagulation cascade pathways and related extracellular matrix and inflammatory signaling pathways (Fig. 3C).

### Western blot validation

**Fig. 4:**
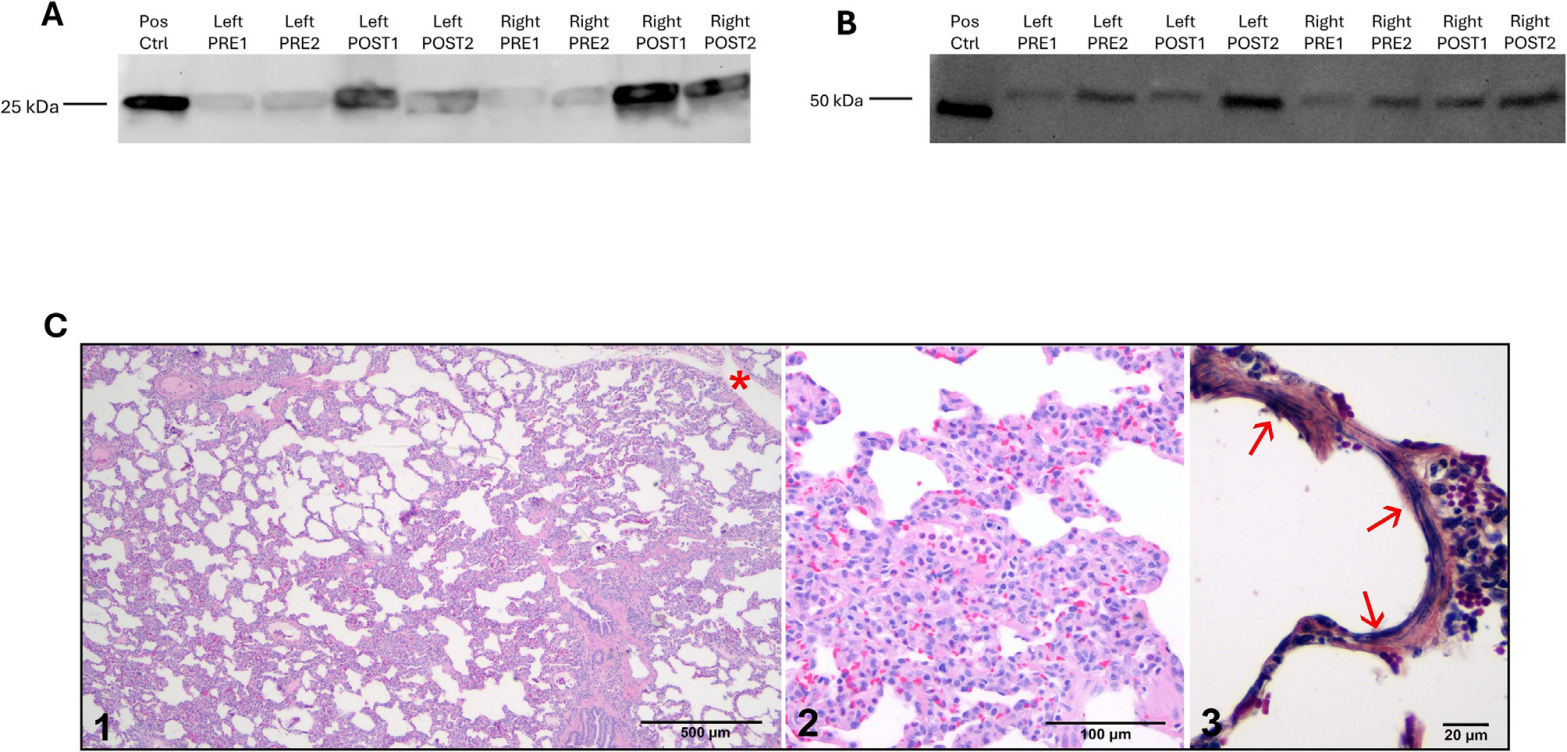
**Validation of proteomic findings by western blot and representative histopathology of lung injury after cardiopulmonary resuscitation**. Panel A shows western blot analysis of apolipoprotein A1 (APOA1, ∼25 kDa) in bronchoalveolar lavage fluid from left and right lungs before (PRE) and after (POST) CPR, demonstrating increased signal intensity in post-CPR samples compared with pre-CPR samples. Panel B shows western blot analysis of serpin family E member 1 (SERPINE1, plasminogen activator inhibitor-1 [PAI-1], ∼50 kDa), also showing increased abundance after CPR in both lungs. Positive controls (Pos Ctrl) were included for both proteins. Panel C shows representative histologic findings from post-CPR lung tissue. Image 1 demonstrates multifocal alveolar collapse and interstitial thickening with regional inflammatory infiltration (asterisk). Image 2 shows higher magnification of alveolar septal thickening with inflammatory cell infiltration and congestion. Image 3 shows phosphotungstic acid hematoxylin (PTAH) staining highlighting fibrin deposition within the alveolar space and septal structures (arrows).

Western blot analysis was performed to confirm selected findings from the proteomic analysis. BALF samples were pooled by lung and time point, and equal amounts of total protein were analyzed for each condition. Compared with pre-CPR samples, apolipoprotein A-I (APOA1) and plasminogen activator inhibitor-1 (PAI-1) showed increased band intensity after CPR in both lungs (Fig. 4A, B). Densitometric analysis using ImageJ demonstrated that APOA1 intensity increased approximately three-fold in the left lung and nearly seven-fold in the right lung, whereas PAI-1 intensity increased by approximately 1.5-fold in both lungs. These results were consistent with the quantitative proteomic analysis and confirmed an increased abundance of these proteins in BALF following resuscitation.

### Histologic findings

Qualitative histologic examination performed in a subset of animals (*n* = 3) showed multifocal lung injury in post-CPR samples (Fig. 4C 1–3). Approximately 30–40% of the examined parenchyma showed expansion of alveolar septa by inflammatory infiltrates composed predominantly neutrophils, with associated vascular neutrophilia. Several alveoli contained thin eosinophilic layers of proteinaceous material mixed with cellular debris, and subpleural and perivascular regions showed mild edema. Phosphotungstic acid hematoxylin (PTAH) staining revealed patchy fibrin deposition along alveolar walls, consistent with acute pneumocyte injury and local fibrin deposition.

## Discussion

Proteomic analysis of BALF obtained before and after cardiopulmonary resuscitation demonstrated significant alterations consistent with early lung injury in this swine model. Differentially abundant proteins identified in both lungs were enriched in pathways related to coagulation and fibrinolysis, complement activation, extracellular matrix organization, and inflammatory responses, as shown by pathway and protein–protein interaction analyses. Increased levels of two of the most highly expressed proteins, PAI-1 and APOA1, were confirmed by western blot, and histologic evaluation showed early structural abnormalities after resuscitation. The overall proteomic profile supports the presence of early barrier dysfunction and inflammatory activation after resuscitation rather than a purely hydrostatic process.

Increased coagulation and antifibrinolytic proteins were a prominent feature of the proteomic response after resuscitation. Multiple proteins involved in fibrinolysis inhibition, extracellular matrix regulation, and protease control were increased in both lungs, including PAI-1, inter-alpha inhibitor family proteins, vitronectin, thrombospondin-4, and complement components. Increased PAI-1 is consistent with suppression of fibrinolysis, a mechanism commonly observed in acute lung injury in which reduced plasmin activity favors fibrin deposition and persistence of intra-alveolar proteinaceous material.^10^, ^19^ In addition to inhibiting plasmin generation, PAI-1 regulates cell adhesion, migration, and tissue repair through interactions with vitronectin and integrin-associated signaling pathways, linking coagulation abnormalities to inflammatory and remodeling responses.^16^ Vitronectin and thrombospondin-4 are matricellular proteins that modulate leukocyte recruitment, endothelial activation, and extracellular matrix organization, and increased expression of these proteins has been associated with vascular inflammation, integrin signaling, and tissue remodeling in experimental lung injury.^13^, ^14^, ^20^, ^21^, ^22^ Inter-alpha inhibitor proteins further regulate protease activity and complement activation and have been implicated in the host response to sepsis and acute lung injury, and ischemic stroke models.^17^, ^23^, ^24^, ^25^ The combined increase in these proteins indicates activation of a coordinated coagulation–fibrinolysis–matrix regulatory network that likely reflects both ongoing barrier injury and inflammatory cell recruitment, as well as early endogenous responses involved in tissue protection and repair during the initial phase of lung injury.^22^, ^26^

Apolipoproteins, particularly APOA1, were among the most increased proteins after resuscitation and may reflect an endogenous protective response to lung injury. APOA1 and related HDL-associated proteins have anti-inflammatory, antioxidant, and endotoxin-neutralizing properties, and APOA1 mimetic peptides have been shown to reduce lipopolysaccharide-induced inflammatory responses, neutrophil recruitment, and cytokine production in experimental lung injury.^18^, ^27^, ^28^, ^29^, ^30^, ^31^, ^32^ In the present study, increased APOA1 after resuscitation may represent a compensatory response to oxidative stress, epithelial injury, and innate immune activation, nonspecific protein leakage into the alveolar space, or both. Regardless of its origin, its precise mechanistic significance requires further study.

Proteins associated with epithelial injury, oxidative stress, and barrier dysfunction were also altered after resuscitation, further supporting the presence of early structural lung damage. Soluble RAGE, a marker of alveolar type I epithelial cell injury, was increased in both lungs, although it was not among the most highly expressed proteins in the shared dataset. Because sRAGE has been extensively validated as a marker of epithelial injury and severity in experimental and clinical ARDS, its increase remains biologically relevant and supports the presence of permeability-type lung injury.^15^, ^33^, ^34^, ^35^, ^36^ Increased extracellular histones were also detected, consistent with release of damage-associated molecular patterns during tissue injury, which have been shown to induce endothelial and epithelial permeability, promote inflammatory activation, and contribute to organ dysfunction in critical illness.^11^, ^12^ In addition, glutathione peroxidase 3 (GPX3), an extracellular antioxidant enzyme involved in protection against oxidative stress, was increased, consistent with activation of redox-regulatory pathways during early lung injury and systemic inflammation.^37^ Lumican, an extracellular matrix proteoglycan elevated in experimental and clinical ARDS, was also increased in both lungs.^38^, ^39^ Lumican has been implicated in neutrophil migration, inflammatory signaling, and early fibrotic responses after lung injury, and its presence further supports activation of remodeling pathways occurring in parallel with epithelial damage.^38^, ^39^, ^40^, ^41^, ^42^

Several proteins involved in epithelial barrier function and host defense were decreased after CPR, including PIGR, CADM1, ENPP1, and DMBT1. PIGR is responsible for transepithelial transport of IgA and IgM and plays an important role in airway mucosal immunity,^43^ while DMBT1 participates in innate immune responses at epithelial surfaces and has been implicated in surfactant-related processes in the injured lung.^44^ CADM1 is involved in epithelial cell adhesion,^45^ and ENPP1 has been linked to epithelial barrier formation and wound healing responses.^46^ In contrast, reduced C5 may reflect complement consumption after activation, as excessive C5a signaling has been associated with inflammatory activation, apoptosis, and propagation of acute lung injury.^47^

The coexistence of epithelial injury markers, oxidative stress responses, and matrix-related proteins suggests that disruption of the alveolar–capillary barrier occurs early after resuscitation and is consistent with mechanisms described in experimental and clinical acute lung injury rather than changes explained solely by hydrostatic edema.

While lung injury and ARDS are increasingly recognized complications after CPR, the molecular mechanisms underlying this process remain poorly defined. Patients who achieve ROSC frequently develop pulmonary dysfunction as part of PCAS, a systemic inflammatory and ischemia–reperfusion state characterized by oxidative stress, coagulopathy, endothelial injury, and multiorgan dysfunction, with the lung being particularly susceptible due to aspiration, pulmonary contusion from chest compressions, infection, and reperfusion injury.^1^, ^4^, ^5^, ^7^, ^48^ Clinical studies have shown that a substantial proportion of patients after cardiac arrest exhibit impaired oxygenation with PaO_2_/FiO_2_ ratios consistent with acute lung injury, and many require mechanical ventilation using lung-protective strategies similar to those used in ARDS, supporting the concept that lung injury after resuscitation shares pathophysiologic features with ARDS. In this context, the exact relevance of the above-listed proteins remains to be determined; however, several of the proteins identified in the present dataset have previously been associated with clinically meaningful outcomes in patients with acute lung injury, ARDS, and sepsis. Plasma levels of sRAGE, a marker of alveolar type I epithelial injury, have been shown in large ARDS Network cohorts to correlate with severity of lung injury and to predict mortality, with higher baseline plasma sRAGE associated with increased risk of death as well as fewer ventilator-free days and fewer organ-failure-free days in patients enrolled in randomized trials of mechanical ventilation for acute lung injury.^15^, ^34^, ^36^ In trauma and critical illness, early elevation of circulating sRAGE also correlates with the extent of parenchymal lung injury quantified by computed tomography and identifies patients with more severe lung damage shortly after admission.^33^, ^35^ Alterations in coagulation and fibrinolytic pathways, including increased PAI-1, have likewise been linked to worse outcomes in acute lung injury, where elevated PAI-1 activity in plasma and BALF is associated with impaired fibrinolysis, increased intra-alveolar fibrin deposition, and higher mortality, supporting a role for dysregulated protease–antiprotease balance in persistent lung injury.^19^, ^49^, ^50^ Consistent with this concept, reduced urokinase-dependent fibrinolytic activity within the alveolar compartment has been described in patients with ARDS and correlates with severity of respiratory failure. Proteins involved in extracellular matrix regulation and fibroproliferative signaling also show prognostic associations, as Lumican has been reported to be elevated in patients with ARDS and to promote early fibrotic responses after lung injury, linking matrix remodeling to the development of persistent lung dysfunction.^38^ In addition, circulating inter-alpha inhibitor proteins, which regulate inflammation and protease activity, decrease in severe sepsis, and lower plasma levels have been associated with more severe illness and increased mortality, supporting their role as biomarkers of systemic inflammatory burden in critically ill patients.^26^, ^51^

A recent translational study by Magliocca and colleagues described cardiopulmonary-resuscitation–associated lung edema (CRALE), emphasizing pulmonary edema as a predominantly hydrostatic phenomenon related to intrathoracic pressure swings during chest compressions.^6^ This interpretation differs from the present findings, which showed protein-rich alveolar fluid and histologic evidence of early injury, suggesting that mechanisms beyond hydrostatic loading may be involved. A subsequent multimodal study by the same group confirmed CRALE after prolonged CPR, with reduced lung compliance, increased lung weight, reduced aeration, and a high wet-to-dry ratio.^52^ Importantly, its histology showed alveolar hemorrhage and tissue replacing airspace, which the authors linked to increased vascular permeability, indicating injury beyond purely hydrostatic edema and complementing our molecular findings. Several methodological differences preclude direct comparison between studies. In the CRALE study, lung abnormalities were more pronounced with mechanical compared with manual compressions, whereas only mechanical compressions were used in the present model. In addition, both no-flow and CPR durations were substantially longer in this study, which may have resulted in greater ischemia–reperfusion injury. Another important difference is the timing and scope of tissue assessment, as the original CRALE study evaluated histology at 72 h after ROSC and did not observe major inflammatory changes,^6^ whereas the later multimodal study, like ours, sampled shortly after resuscitation and identified hemorrhage and tissue injury.^52^ In the present study, histologic evaluation was likewise performed shortly after ROSC, although in a limited number of animals. It is therefore possible that the molecular and histologic abnormalities observed here represent an early phase that could partially resolve over time. Whether these early permeability-associated changes are related to the subsequently described CRALE phenotype remains to be determined. These differences highlight the need for further studies in translational models and in humans to determine the temporal evolution of lung injury after CPR and to establish whether early molecular markers are associated with the subsequent trajectory of pulmonary dysfunction and clinical outcomes.

This study has several limitations. The sample size was small, and histologic evaluation was qualitative and descriptive, performed by a blinded pathologist in a limited number of animals (*n* = 3) and in post-CPR tissue only, without baseline or control tissue and without a standardized lung injury score. The histologic findings should therefore be regarded as illustrative support for the proteomic data rather than a quantitative assessment of injury. Measurements were obtained at a single early time point after ROSC, without longitudinal follow-up, and therefore the temporal evolution of the observed molecular and histologic changes cannot be determined. In addition, post-CPR BALF was obtained at different physiological states, immediately after ROSC in resuscitated animals and at the end of CPR in those that were not. However, no-flow duration and the resuscitation protocol were standardized, perfusion and ventilation surrogates during the sampling window were comparable between groups, and the direction of change of the shared differentially abundant proteins was consistent across subgroups, arguing against ROSC status as the primary driver of the observed signature. The proteomic analysis was descriptive and hypothesis-generating, and the functional relevance of the identified proteins cannot be established. In addition, BALF cannot distinguish local production, release from injured resident cells, alveolar-capillary barrier leak, and plasma contamination. Recovery was consistent across samples, but we did not measure albumin, IgM, urea, or paired plasma, so protein source and dilution cannot be fully resolved. We therefore describe over-representation of complement and coagulation proteins in the alveolar compartment rather than proven local activation. Even so, entry of plasma proteins into the alveolar space reflects a change at the alveolar-capillary interface, and a protein-rich fluid is more consistent with increased permeability than with hydrostatic edema alone. No sham or time-matched mechanically ventilated control group without cardiac arrest and CPR was included, so the independent contributions of anesthesia, instrumentation, repeated bronchoscopy and lavage, mechanical ventilation, inspired oxygen, time, hemodynamic instability, cardiac arrest, ischemia and reperfusion, and chest compressions cannot be separated, and the present findings should be regarded as associated with, rather than caused by, cardiopulmonary resuscitation. In particular, the inflammatory and permeability-associated changes may also reflect hyperoxia, ischemia–reperfusion, and activation of circulating neutrophils and mediators during the arrest and resuscitation period, rather than the mechanical effect of chest compressions alone. We note, however, that in this paired design each animal served as its own baseline, and that the pre-arrest sample was obtained only after approximately three to four hours of anesthesia, ventilation, instrumentation, recumbency, and an initial gently performed bronchoscopy. Because these slower background exposures were already present at baseline, they are unlikely on their own to explain the marked change in alveolar protein composition that emerged over the subsequent 45–55 min spanning arrest and resuscitation. Only mechanical chest compressions were used, and the relatively prolonged no-flow and CPR durations in this model may not reflect all clinical scenarios of cardiac arrest. Western blot was limited to two proteins and used samples pooled by time point and lung, so it confirmed the direction of change rather than providing animal-level statistics, which came from the paired per-animal proteomic data. Individual-sample western blot or ELISA would strengthen validation. Pathway enrichment is exploratory and depends on database annotation, which may flag pathways by shared protein membership rather than specific mechanisms.

In conclusion, early after resuscitation from cardiac arrest, the alveolar compartment showed proteomic changes, accompanied by qualitative histologic evidence of inflammation and edema, consistent with barrier dysfunction. These results support the concept that early post-CPR pulmonary abnormalities may reflect a mixed hydrostatic–permeability process rather than purely pressure-related edema. If confirmed in larger and longitudinal studies, early molecular changes in the alveolar compartment may help identify biomarkers of lung injury after cardiac arrest and improve understanding of the mechanisms that determine the progression from transient edema to more severe inflammatory lung dysfunction. Future studies should also determine whether less invasive measurements, such as plasma concentrations of selected proteins, correlate with pulmonary findings and may serve as clinically useful biomarkers after resuscitation.

## CRediT authorship contribution statement

**Joaquin Araos:** Writing – review & editing, Writing – original draft, Supervision, Investigation, Formal analysis, Data curation, Conceptualization. **Felipe Teran:** Writing – review & editing, Writing – original draft, Methodology, Investigation, Formal analysis, Data curation, Conceptualization. **Clark Owyang:** Writing – review & editing, Writing – original draft, Visualization, Methodology, Investigation, Formal analysis, Data curation, Conceptualization. **Derek Lao:** Writing – review & editing, Methodology, Data curation. **Congli Zeng:** Writing – review & editing, Methodology, Formal analysis. **Marcos Vidal Melo:** Writing – review & editing, Methodology, Formal analysis. **Qin Fu:** Writing – review & editing, Formal analysis. **Rory C. Chien:** Writing – review & editing, Methodology, Formal analysis. **Michael Garenani:** Methodology, Investigation, Formal analysis. **Manuel Martin-Flores:** Writing – review & editing, Methodology, Investigation, Data curation, Conceptualization. **Marta Cercone:** Writing – review & editing, Supervision, Investigation, Formal analysis, Conceptualization.

## Ethics approval and consent to participate

All experimental procedures were reviewed and approved by the Institutional Animal Care and Use Committee of Cornell University (protocol 2022-0135) before study initiation and were conducted in accordance with ARRIVE guidelines and institutional regulations for animal research.

## Consent for publication

Not applicable.

## Availability of data and materials

All data supporting the findings of this study are available within the article and its supplementary files. Additional information can be made available from the corresponding author upon reasonable request.

## Funding

This study was supported by a Multi-Investigator Seed Grant from Cornell University.

## Authors' contributions

JA, FT, CO, MMF and MC designed the study, conducted the experiments, analyzed the data, and wrote the manuscript. DL participated in the experimental procedures, prepared samples for proteomic analysis, and reviewed the manuscript. CZ and MVM analyzed the proteomic results, interpreted the data, and contributed to manuscript writing. QF performed the proteomic analyses, generated the proteomic figures, and conducted the associated statistical analyses. RC and MG performed histologic analysis, contributed to data interpretation, and assisted in manuscript preparation. All authors reviewed and approved the final manuscript.

## Declaration of competing interest

Felipe Teran is a recipient of National Institutes of Health/National Heart, Lung, and Blood Institute grant K23 HL165150. Felipe Teran is the owner of ResusMedX LLC, Course Director of the Resuscitative TEE Workshop, and Chair of the Scientific Oversight Committee of the Resuscitative TEE Collaborative Registry (rTEECoRe) Network. Clark Owyang is a recipient of a Zoll Foundation Award. Clark Owyang is part of the Course Faculty for the Resuscitative TEE Workshop and a Member of the Scientific Oversight Committee of the Resuscitative TEE Collaborative Registry (rTEECoRe) Network. Joaquin Araos is a recipient of a Zoll Foundation Award. Joaquin Araos is also the recipient of two internal Cornell University College of Veterinary Medicine awards, the Resident Research Award and The Harry M. Zweig Memorial Fund for Equine Research, as well as a United States Department of Defense award (HT9425-25-1-0142). Marcos Vidal Melo is funded by National Institutes of Health grants NHLBI R01 HL121228 and UH3 HL140177. All other authors declare no conflicts of interest.

## Appendix A Supplementary material

Supplementary File 1 Supplementary Methods and Figures. Detailed experimental methods, representative physiologic recordings during the CPR model, and principal component analysis of bronchoalveolar lavage fluid proteomes by ROSC status.

Supplementary File 2 Supplementary Proteomics Dataset. Complete quantitative bronchoalveolar lavage fluid proteomics dataset, including normalized protein abundances, differential abundance analysis, and functional enrichment results for the left and right lungs.

Supplementary Data 1 Supplementary Western Blot Images. Uncropped Western blot images for APOA1 and PAI-1 with corresponding total protein staining used for validation of proteomic findings
