# Supplementary material for: Early alveolar molecular signatures after cardiopulmonary resuscitation: a bronchoalveolar lavage (BALF) proteomic study in swine
Source: Resusc Plus. 2026 Jul 6;30:101405. doi: 10.1016/j.resplu.2026.101405 (PMC13427483; doi:10.1016/j.resplu.2026.101405)
Supplement: Supplementary File 1 — Supplementary Methods and Figures. Detailed experimental methods, representative physiologic recordings during the CPR model, and principal component analysis of bronchoalveolar lavage fluid proteomes by ROSC status. [file mmc1.docx]

**Early Alveolar Molecular Signatures After Cardiopulmonary Resuscitation: A Broncho-alveolar Lavage (BALF) Proteomic Study**

**Supplementary File 1.**

Materials and Methods

Animals.

All procedures were conducted in accordance with the ARRIVE guidelines and were approved by the Institutional Animal Care and Use Committee (protocol 2022-0135). [1] Nine juvenile Yorkshire-cross pigs (5 males, 4 females, 30–40 kg) were fasted for 12 hours with free access to water prior to the experiment. Animals were premedicated with ketamine (5 mg/kg), dexmedetomidine (0.04 mg/kg), and midazolam (0.1 mg/kg) intramuscularly. After sedation, a catheter was placed in the marginal ear vein and anesthesia was induced intravenously with propofol to effect.

Animals were intubated with a cuffed endotracheal tube (6.0–7.0 mm internal diameter) and mechanically ventilated (Dräger Evita XL®, Lübeck, Germany) in volume-controlled mode with a tidal volume of 8 mL/kg, respiratory rate 12–14 breaths/min, positive end-expiratory pressure of 5 cmH_2_O, and inspired oxygen fraction of 0.6. Intravenous fluids were administered at 5 mL/kg/h, and morphine (0.1 mg/kg IV) was given prior to instrumentation. Pulse oximetry, capnography, electrocardiography, invasive arterial pressure, and core temperature were continuously monitored, and animals were maintained in the supine position throughout the experiment.

Experimental protocol.

The present experiment was performed within an established large-animal model of cardiac arrest designed for detailed physiologic monitoring during resuscitation. An 8F introducer sheath was placed in the right external jugular vein. An 8F introducer sheath was placed in the right external jugular vein. A three-lumen pulmonary artery catheter (Swan-Ganz, Edwards Lifesciences, Irvine, CA, USA) was advanced into the pulmonary artery. The mid port was positioned in the right ventricle, confirmed by the characteristic right ventricular pressure waveform. A pacing wire (5-Fr bipolar pacing catheter, Edwards Lifesciences, Irvine, CA, USA) was then introduced through the mid lumen of the catheter and advanced until ventricular ectopy was observed, after which ventricular fibrillation (VF) was induced using a 9-V battery as previously described. [2]

After induction of VF, no chest compressions or ventilations were delivered for 8 minutes to simulate a no-flow interval following cardiac arrest. At minute 8, cardiopulmonary resuscitation (CPR) was initiated with mechanical chest compressions and positive-pressure ventilation. During CPR, ventilation was continued in volume-controlled mode with a tidal volume of 8 mL/kg, a respiratory rate of 10 breaths/min, an inspired oxygen fraction of 1.0, a positive end-expiratory pressure of 5 cmH_2_O, and an inspiratory-to-expiratory ratio of 1:2, delivered asynchronously to chest compressions.

Compressions were delivered using a fully adjustable mechanical compression device (Life-Stat 1008, Michigan Instruments, Kentwood, MI) at a rate of 100 compressions/min and a depth of approximately 5 cm, corresponding to one third of the anterior–posterior chest diameter. During the first 5 minutes of CPR, no drugs or defibrillation were administered to reproduce a basic life support phase. After 5 minutes, advanced cardiac life support was initiated, including epinephrine (0.01 mg/kg IV) every 3 minutes. After 10 minutes of CPR, a 200-J biphasic defibrillation attempt was performed. CPR was continued until return of spontaneous circulation (ROSC) or for a maximum of 45 minutes, after which the experiment was terminated.

Broncho-alveolar lavage.

Broncho-alveolar lavage (BAL) samples were collected before inducing cardiac arrest and within 45 minutes following cardiac arrest and CPR, or immediately after return of spontaneous circulation when it occurred. BAL samples were collected via flexible endoscope passed through the tracheal tube. High definition videoendoscope (5 mm outer diameter, 60 cm length, Ambu® aScope^TM^, Ambu, Columbia MD, USA) was placed through the free port of a three-way adaptor into the lumen of the tracheal tube and advanced for its whole length in the right and left caudal lobe (randomized order). Once the endoscope was seeded within the smaller bronchi of the caudal lobe, ventilation was withheld, and warm saline (2ml/kg/lobe) was infused in the lung through the instrument channel and then retrieved. The recovered fluid was placed over ice immediately. BAL fluid was centrifuged at 320 × g for 10 min at 4°C to remove the cellular fraction. The supernatant was centrifuged again at 500 x g for 10 min at 4°C to obtain the final sample that was added protease inhibitor cocktail (10 ul of both Protease Inhibitor and 0.5M EDTA for each ml of BAL fluid). Samples were then stored at −80 °C until analysis.

Protein quantitation.

Protein concentration of each sample (18 pig samples total, 9 pre-CPR and 9 post-CPR) was determined by running a precast 10% Bis-Tris mini-gel with 26-well from Bio-Rad (Hercules, CA) along with a series of amounts of *E. coli* lysates (0.5,1, 2.5, 5, 10, 15 µg/lane). The SDS gel was visualized with Colloidal Coomassie staining (Invitrogen), imaged by ChemDoc imager and analyzed by Image Lab 6.1 software (Bio-Rad) for protein quantitation.

Protein digestion and TMT18-plex labeling.

Further processing of the proteins was then performed according to Thermo Scientific’s TMT Mass Tagging Kits and Reagents protocol (<http://www.piercenet.com/instructions/2162073.pdf> ). A total of 15 µg protein of each sample for 18 samples in the final buffer concentration: 50mM triethylammonium bicarbonate (TEAB) pH 8.5, 6M Urea, 2M Thiourea, 2% SDS. All samples were reduced with 10 mM tris(2-carboxyethyl)phosphine for 1 h at 34 °C, alkylated with 23 mM iodoacetamide for 1 h in the dark and then quenched with a final concentration of 27 mM Dithiothreitol (DTT). Each sample was digested separately using the S-Trap Micro Spin column (Protifi, Huntington NY). [3] After quenching, 12% phosphoric acid was added to a final concentration of 1.2%, followed by 1:7 dilution (v/v) with 90% methanol, 0.1M TEAB pH 8.5. The samples were then placed into individual spin columns and centrifuged 4000g for 30 sec. The spin columns were washed three times with 400 µl 90% methanol, 0.1 M TEAB pH 8.5. Digestion was performed with 150 µl trypsin at 10 ng/µl (1:10 w/w) in 50 mM TEAB pH 8.5 added to the top of the spin columns that were incubated overnight (16 hr) at 37 °C. Following incubation, the digested peptides were eluted off the S-trap column sequentially with 80 µl each of 50 mM TEAB pH 8.5 followed by 0.2% formic acid and finally 50% acetonitrile, 0.2% formic acid. Three eluted peptides fractions were pooled together and dried in speed vac. All dried samples were resuspended with 100 µl Optima water and re-dried to ensure the complete removal of formic acid. Immediately before labeling, each sample was reconstituted in 100 µl 0.1M TEAB pH 8.5. The TMT 18-plex labels (0.5 mg dried powder) were reconstituted with 50 µL of anhydrous ACN prior to labeling and added with 1: 2 ratio to each of the 100 µl tryptic digest samples and incubated at 1 hour at room temperature. The labeled peptides from the samples were pooled together. The pooled peptides of each set were then evaporated to dryness and subjected to cleanup by solid phase extraction (SPE) on MCX Cartridges (Waters, Milford, MA). A small aliquot of pooled sample was checked for successful incorporation of the TMT label to each sample using Orbitrap Fusion (Thermo-Fisher Scientific, San Jose, CA). The rest pooled samples were subjected to the first dimensional LC fractionation via a high pH reverse phase chromatography.

High pH Reversed-Phase (HpRP) Peptide Fractionation.

The HpRP liquid chromatography was carried out using a Dionex UltiMate 3000 HPLC system with the built-in micro fraction collection option in its autosampler and UV detection (Thermo Scientific, Sunnyvale, CA). Specifically, the TMT 18-plex tagged tryptic peptides were reconstituted in buffer A (20 mM ammonium formate pH 9.5 in water), filtered by 0.22μm cellulose acetate filter (Costar Spin-X, Corning Inc., Salt Lake City, UT) and loaded onto an XBridge C18 column (3.5 µm, 2.1x 150 mm) from Waters, (Milford, MA) with 20 mM ammonium formate (NH_4_FA), pH 9.5 as buffer A and 85% ACN/15% 20 mM NH_4_FA as buffer B. The LC was performed using a linear gradient: 5-5-14-50-90-90-5-5 %B, 0-0.25-1-40-42-45-45.5-49 min. at a flow rate 200 µL/min. The column temperature was maintained at 30 °C and the collection plate at 5 °C. Forty-eight fractions were collected at 1-minute intervals and pooled into a total of 6 fractions based on the UV absorbance at 214 nm and with multiple fraction concatenation strategy. [4] Each of the 6 fractions was dried and reconstituted in 56 µL of 2% ACN/0.5% FA for nanoLC-MS/MS analysis.

Nano-scale reverse phase chromatography and tandem MS (nanoLC-MS/MS).

The nanoLC-MS/MS analysis was carried out using an Orbitrap Eclipse (Thermo-Fisher Scientific, San Jose, CA) mass spectrometer equipped with a nanospray Flex Ion Source coupled with the UltiMate 3000 RSLCnano (Dionex, Sunnyvale, CA). Each reconstituted fraction (3.5 μL = 0.7 µg for global proteomics fractions) was injected onto a PepMap C-18 RP nano trap column (5 µm, 100 µm × 20 mm, Dionex) at 20 μL/min flow rate for rapid sample loading, and separated on a PepMap C-18 RP nano column (2 µm, 75 µm x 25 cm). The column was equilibrated with 2% acetonitrile (ACN) in 0.1% aqueous formic acid (eluant A) prior to each run. The labeled peptides were eluted in a 120-min gradient of 5% to 33% eluant B containing 95% ACN in 0.1% formic acid at 300 nL/min, followed by an 8-min ramping to 90% B, a 7-min hold and 21-min re-equilibration with 2% ACN-0.1% FA prior to the next run. The Orbitrap Eclipse was operated in positive ion mode with nano spray voltage set at 1.9 kV and source temperature at 300 °C. External calibration for FT, IT and quadrupole mass analyzers was performed. Raw MS data files for all the fractions were acquired using a real-time search (RTS) synchronous precursor selection (SPS) MS^3^ workflow as reported previously. [5] Specifically, the RTS MS^3^ workflow consisted of 2.5 second “Top Speed” data-dependent CID-MS/MS scans (for peptide identifications by RTS) that enabled to trigger SPS of 10 MS^2^ product ions for subsequent MS^3^ in FT. In RTS node, the *Sus scrofa* Uniprot FASTA database containing 46,175 sequences was imported along with trypsin as the enzyme for real-time spectral database search for the samples from corresponding species. The search parameters included: TMTpro modification on N-terminal amines (∆mass 304.2071) and carbamidomethyl modification of cysteine (∆mass 57.0215) as static modifications, TMTpro modification (∆mass 304.2071) on lysine as variable modifications, maximum 3 variables per peptide, and 2 maximum missed cleavage allowed. A maximum search time for 35 ms was allowed for the RTS MS^3^ searching. The MS^3^ scan was carried out using a mass range of 110-500 m/z, an MS isolation window of 1.1 m/z and MS^2^ isolation window of 2.0 m/z were used. A resolving power of 50,000 at MS^3^ with a normalized collision energy of 55% was used for peptide quantitation. Other parameters included 200% normalized AGT target and 120 ms for maximum injection time. Dynamic exclusion parameters were set at 1 count within 50s exclusion duration with ±10 ppm exclusion mass window. All data were acquired under Xcalibur 4.4 operation software in Orbitrap Eclipse (Thermo Fisher Scientific, San Jose, CA).

Data processing, protein identification and data analysis.

All raw MS spectra were processed and searched using the Sequest HT search engine within the Proteome Discoverer 2.5 (PD2.5, Thermo). The same database for pigs used for RTS data acquisition as described above was used for post-MS database searches. The default search settings used for TMT 18-plex quantitative processing and protein identification in PD2.5 searching software were: two mis-cleavage for full trypsin with fixed carbamidomethyl modification of cysteine, fixed 18-plex TMT modifications on lysine and N-terminal amines along with variable modifications of methionine oxidation, deamidation on asparagine/glutamine residues and protein N-terminal acetylation. The peptide mass tolerance and fragment mass tolerance values were 10 ppm for MS survey scan, 0.6 Da for MS^2^ and 20 ppm for MS^3^, respectively. Identified peptides were further filtered for maximum 1% FDR using the Percolator algorithm in PD 2.5 along with additional peptide confidence set to high and peptide mass accuracy ≤5 ppm. The TMT18-plex quantification method within Proteome Discoverer 2.5 software was used to calculate the reporter ion abundances in MS^3^ spectra that were corrected for the isotopic impurities. Both unique and razor peptides were used for relative protein quantitation. Signal-to-noise (S/N) values were used to represent the reporter ion abundance with a co-isolation threshold of 50% and an average reporter S/N (intensity) threshold of ≥10 used for quantitation spectra. The intensities of peptides, which were summed from the intensities of the PSMs, were summed to represent the abundance of the proteins. For relative ratio between the two groups, normalization on sum of total peptide intensities for each sample was applied. The search results including ratio, peptide abundance for each sample were output to Microsoft Excel software for further data analysis. Bronchoalveolar lavage samples obtained from the left and right lungs were processed and analyzed independently. Differential abundance analysis was performed separately for left and right lung datasets by comparing post-CPR versus pre-CPR samples for each lung.

Bioinformatics analysis.

Proteins were considered to be differentially abundant proteins (DAP) if the difference was statistically significant (P < 0.05) by MetaboAnalyst (version 6.0, <https://www.metaboanalyst.ca>) with a fold change >1.5 or <0.67. Differential-abundance p-values were not adjusted for multiple comparisons; instead, robustness was supported by requiring concordance between the two independently analyzed lungs, and the differentially abundant protein list is considered hypothesis-generating. The protein-protein interaction (PPI) network was visualized using the STRING database (version 11.0, http://string-db.org). The DAVID functional annotation tool (http://david.abcc.ncifcrf.gov) was used to verify gene ontology (GO) and pathway databases to find enriched categories. The interaction network of the corresponding pathways and proteins was constructed by Cytoscape (version 3.7.2, <https://cytoscape.org/>).

To identify the most consistent molecular changes associated with resuscitation, differentially abundant proteins identified in the left and right lungs were compared, and only proteins present in both datasets were retained for downstream analyses. Heatmap visualization, protein-protein interaction analysis, gene ontology enrichment, and pathway analysis were performed using the overlapping differentially abundant proteins common to both lungs. Principal component analysis and volcano plots were generated separately for left and right lung datasets.

Western Blot Validation.

To confirm selected findings from the proteomic analysis, Western blot was performed for apolipoprotein A-I (ApoA1) and plasminogen activator inhibitor-1 (PAI-1), which were among the most increased proteins identified by quantitative proteomics. BALF samples were pooled by time point (pre-CPR and post-CPR) and by lung (left and right) to obtain sufficient protein for analysis. Equal amounts of total protein were separated by SDS-PAGE and transferred to PVDF membranes. Membranes were incubated with primary antibodies against ApoA1 (rabbit polyclonal, MyBioSource) and PAI-1 (mouse monoclonal, Proteintech), followed by species-appropriate secondary antibodies. Protein bands were visualized using chemiluminescence and quantified by densitometry using ImageJ (National Institutes of Health). Band intensity was normalized to total protein staining, and post-CPR to pre-CPR ratios were calculated for each lung.

Histologic analysis

Lung tissue was obtained from three animals at the end of the experiment for morphologic evaluation. Samples were collected from caudal lung lobes corresponding to the regions used for bronchoalveolar lavage fluid (BALF) sampling. Tissue specimens were fixed in 10% neutral buffered formalin, embedded in paraffin, and sectioned at 4 µm thickness. Sections were stained with hematoxylin–eosin for general histologic evaluation, and phosphotungstic acid hematoxylin (PTAH) staining was performed to assess fibrin deposition. Slides were evaluated by a board-certified veterinary pathologist blinded to sampling time point.

1. Percie du Sert N, Hurst V, Ahluwalia A, Alam S, Avey MT, Baker M, Browne WJ, Clark A, Cuthill IC, Dirnagl U *et al*: **The ARRIVE guidelines 2.0: Updated guidelines for reporting animal research**. *PLoS Biol* 2020, **18**(7):e3000410.

2. Teran F, Owyang CG, Martin-Flores M, Lao D, King A, Palasz J, Araos JD: **Hemodynamic impact of chest compression location during cardiopulmonary resuscitation guided by transesophageal echocardiography**. *Crit Care* 2023, **27**(1):319.

3. Yang Y, Anderson E, Zhang S: **Evaluation of six sample preparation procedures for qualitative and quantitative proteomics analysis of milk fat globule membrane**. *Electrophoresis* 2018, **39**(18):2332-2339.

4. Wang Y, Yang F, Gritsenko MA, Wang Y, Clauss T, Liu T, Shen Y, Monroe ME, Lopez-Ferrer D, Reno T *et al*: **Reversed-phase chromatography with multiple fraction concatenation strategy for proteome profiling of human MCF10A cells**. *Proteomics* 2011, **11**(10):2019-2026.

5. Fu Q, Liu Z, Bhawal R, Anderson ET, Sherwood RW, Yang Y, Thannhauser T, Schroyen M, Tang X, Zhang H *et al*: **Comparison of MS(2), synchronous precursor selection MS(3), and real-time search MS(3) methodologies for lung proteomes of hydrogen sulfide treated swine**. *Anal Bioanal Chem* 2021, **413**(2):419-429.


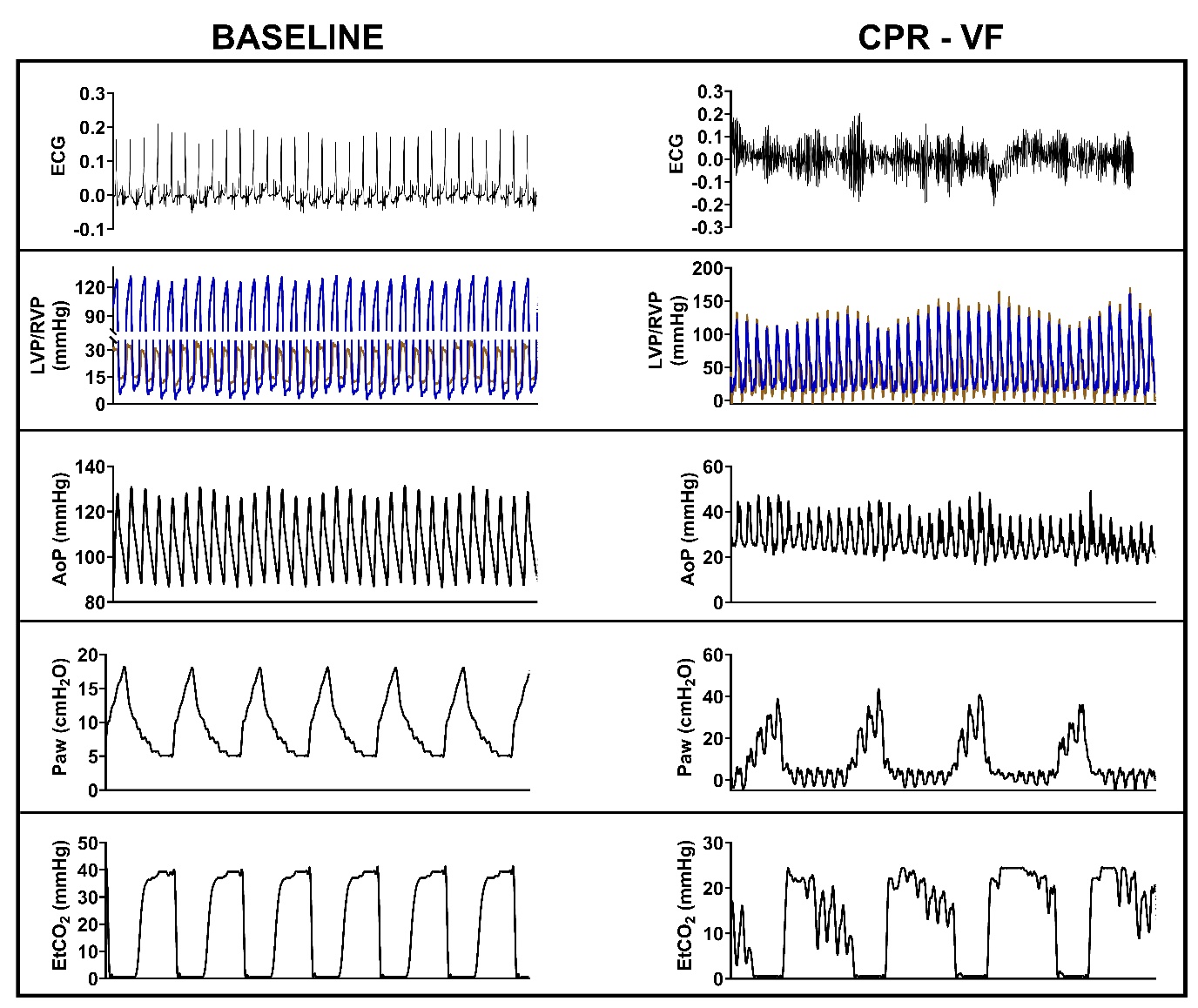


**Supplementary Figure 1. Representative physiologic recordings at baseline and during ventricular fibrillation with cardiopulmonary resuscitation.** Left panels show baseline conditions and right panels show ventricular fibrillation with ongoing cardiopulmonary resuscitation. At baseline, electrocardiogram (ECG) is shown. Left ventricular pressure (LVP, blue) and right ventricular pressure (RVP, orange) were measured using solid-state pressure catheters (Millar Instruments, Houston, TX). Aortic pressure (AoP) was measured at the level of the abdominal aorta using a solid-state catheter (Millar Instruments, Houston, TX). Airway pressure (Paw) reflects volume-controlled ventilation with positive end-expiratory pressure (PEEP) of 5 cmH_2_O and tidal volume (VT) of 8 to 10 mL/kg. End-tidal carbon dioxide (EtCO_2_) is shown. During ventricular fibrillation, ECG is shown. Continuous chest compressions (CC) were delivered at 100 compressions per minute using a mechanical chest compression device (Michigan Instruments, Grand Rapids, MI). LVP and RVP were recorded with Millar catheters. Paw shows positive-pressure ventilation delivered without interruption of chest compressions with PEEP of 5 cmH_2_O, tidal volume of 8 mL/kg, and respiratory rate of 10 breaths per minute. Oscillations observed in Paw and EtCO_2_ reflect the effect of chest compressions delivered at the set rate.


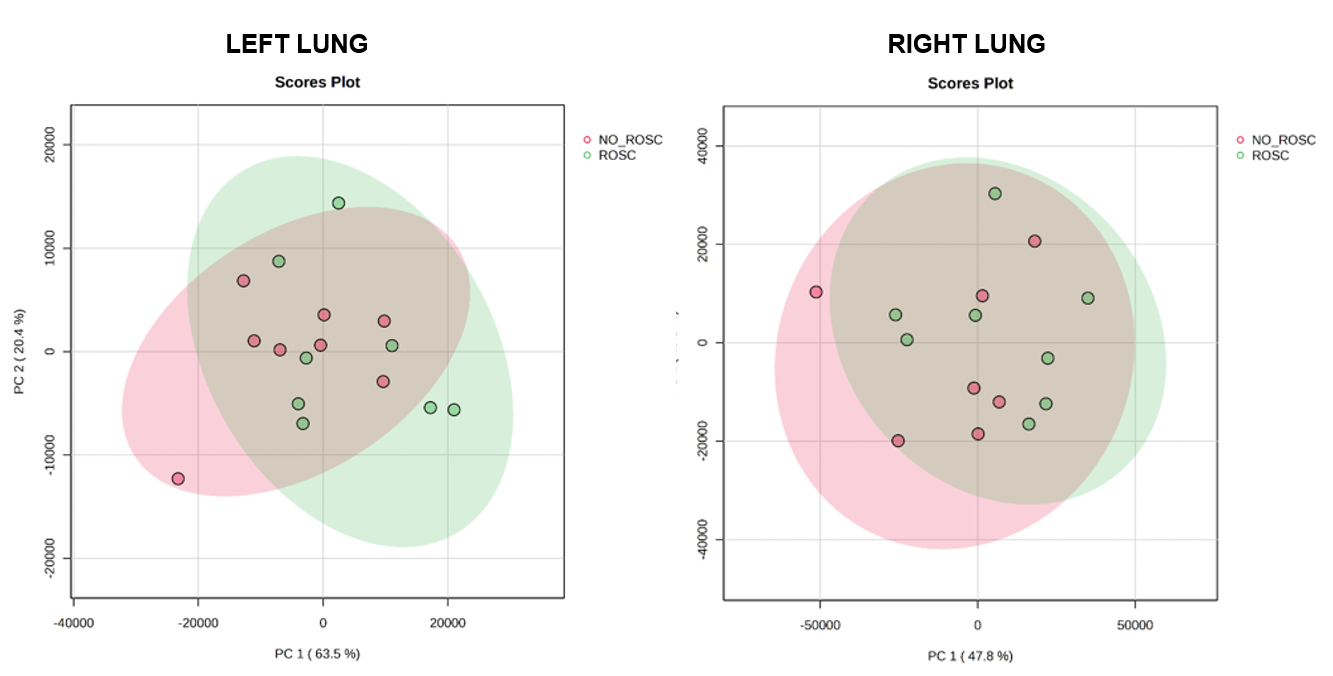


**Supplementary Figure 2.** Principal component analysis (PCA) of bronchoalveolar lavage fluid proteomes by return of spontaneous circulation (ROSC) status. Scores plots are shown for the left lung (left panel) and right lung (right panel), with samples colored by ROSC status (ROSC, green; NO_ROSC, pink) and shaded 95% confidence regions for each group. In both lungs, ROSC and non-ROSC samples overlapped extensively without clear separation, indicating that the differentially abundant protein signature was not driven by ROSC status.
