# Supplementary figures and images for: Early alveolar molecular signatures after cardiopulmonary resuscitation: a bronchoalveolar lavage (BALF) proteomic study in swine
Source: Resusc Plus. 2026 Jul 6;30:101405. doi: 10.1016/j.resplu.2026.101405 (PMC13427483; doi:10.1016/j.resplu.2026.101405)

**Supplementary Figure 1**. APOA1 and Total Protein


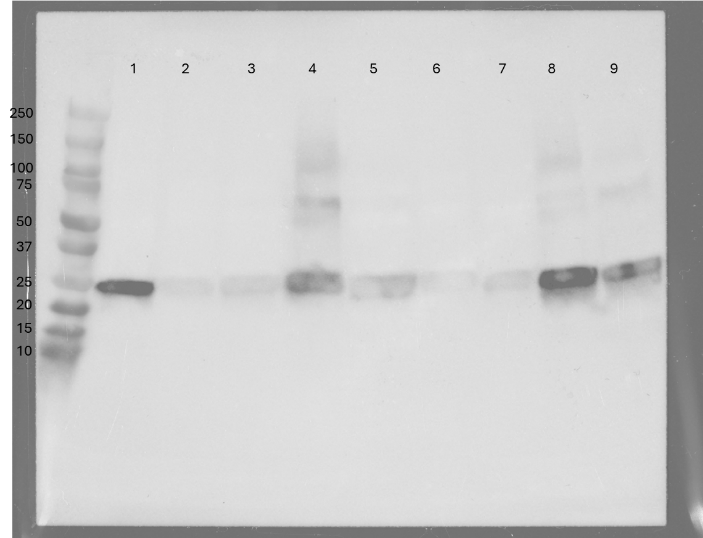

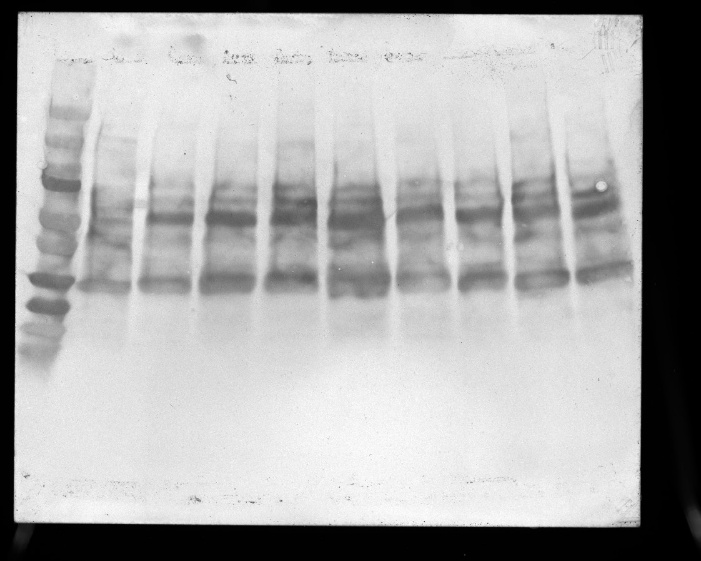


**Supplementary Figure 2**. PAI-1 and Total Protein


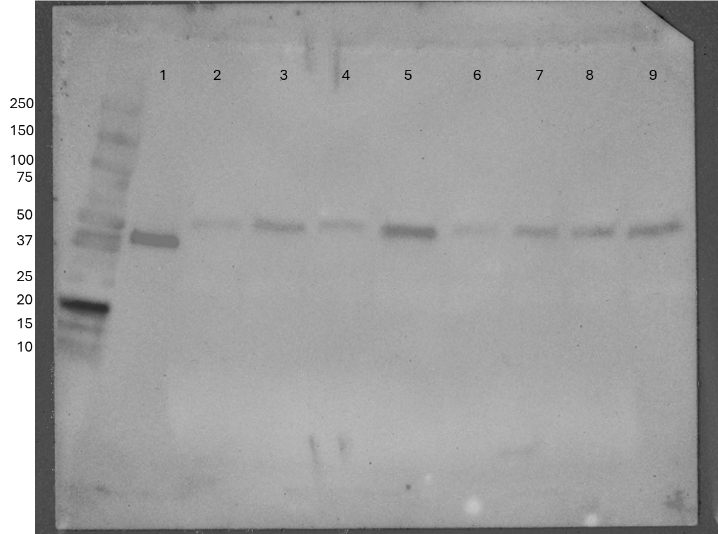

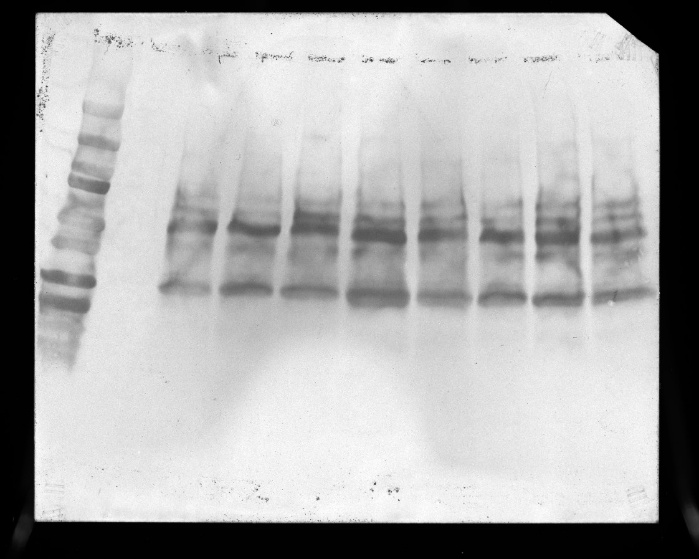

Supplement: Supplementary Data 1 — Supplementary Western Blot Images. Uncropped Western blot images for APOA1 and PAI-1 with corresponding total protein staining used for validation of proteomic findings [file mmc3.docx]
